# The Double Life-Saving Approach of Abdominal Radical Trachelectomy during Pregnancy for Early-Stage Cervical Cancer—An Overview of the Literature and Our Institutional Experience

**DOI:** 10.3390/jpm11010029

**Published:** 2021-01-05

**Authors:** Mihai Stanca, Victoria Ciobanu, Mihai Gheorghe, Szilard Leo Kiss, Alexandra Lavinia Cozlea, Mihai Emil Căpîlna

**Affiliations:** First Obstetrics and Gynecology Clinic, University of Medicine, Pharmacy, Science and Technology “G.E. Palade” of Târgu Mureș, Gheorghe Marinescu Street, Number 38, 540142 Târgu Mureș, Romania; vica.ciobanu@gmail.com (V.C.); mihai18go@gmail.com (M.G.); k.szilardleo@icloud.com (S.L.K.); alexandra.cozlea@gmail.com (A.L.C.); mcapilna@gmail.com (M.E.C.)

**Keywords:** abdominal radical trachelectomy, pregnancy, fertility preservation, cervical cancer, literature search

## Abstract

(1) Background: Cervical cancer is the most common type of cancer encountered during pregnancy, with a frequency of 0.8–1.5 cases per 10,000 births. It is a dire condition endangering patients’ lives and pregnancy outcomes, and jeopardizing their fertility. However, there is a lack of current evidence and consensus regarding a standard surgical technique for pregnant patients who suffer from this condition during pregnancy. The study aims to comprehensively update all published data, evaluating the obstetrical and oncological results of pregnant patients who underwent abdominal radical trachelectomy during early stages of cervical cancer. (2) Methods: A literature search on the Medline, PubMed, and Google Scholar databases was performed, including all articles in question up to July 2020. This study presents an overview of the literature and our institutional experience. (3) Results: A total of 25 cases of abdominal radical trachelectomy were performed during pregnancy for early cervical cancer, including the five cases managed by the authors. Of these, 81% (19 patients) gave birth to live newborns through elective C-section, and 19% (6 patients) experienced miscarriage shortly after the procedure. None of the 25 patients (100%) reported disease recurrence. (4) Conclusions: The results of the current study were satisfactory. However, abdominal radical trachelectomy does not represent the current standard of care for cervical cancer during pregnancy, but it could play an important role if more evidence on its effectiveness will be provided.

## 1. Introduction

Cervical cancer is the fourth most common cancer in females, with approximately 570,000 new cases in 2018, accounting for 6.6% of all cancers in women [1]. It is also the most common type of cancer encountered during pregnancy, with a frequency of 0.8–1.5 cases per 10,000 births [2]. With an increasing number of women delaying reproduction for various reasons, fertility preservation has gained significant importance. Recent advances in screening and early diagnosis [3,4] have allowed for the implementation of conservative management strategies. Radical trachelectomy represents the foundation of conservative surgery and is considered to be a viable option for patients with early-stage cervical cancer (Stages IA2–IB1) who desire fertility preservation [5].

Abdominal radical trachelectomy does not represent the current standard of care for cervical cancer during pregnancy. The recommendations of the Third International Consensus Meeting on Gynecologic Cancers in Pregnancy approve less invasive methods, giving special importance to the use of neoadjuvant chemotherapy after providing adequate information about the possible negative effects on prognosis, pregnancy outcome, and the lack of available data [6]. However, abdominal radical trachelectomy might play an important role in treating this specific category of patients if more evidence on its effectiveness will be provided.

The study aims to comprehensively update all published data, evaluating the obstetrical and oncological results of pregnant patients who underwent abdominal radical trachelectomy during the early stages of cervical cancer.

## 2. Materials and Methods

The study was approved by the Institutional Review Board (IRB) of our institute (ethical approval code: 34535), and written informed consent was obtained from all subjects.

We provide our data for the reproducibility of this study in other centers if such is requested.

In the following paper, we present an overview of the literature on this topic. A thorough search was conducted on the Medline, PubMed, and Google Scholar databases using the following keywords in various combinations: abdominal radical trachelectomy, pregnancy, fertility preservation, cervical cancer, and literature search. All published articles regarding abdominal radical trachelectomy performed during pregnancy for early stages of cervical cancer up to July 2020 were considered. Furthermore, we describe our institutional experience of performing this procedure on five patients.

All patients underwent abdominal radical trachelectomy during pregnancy. Unlike the original abdominal radical trachelectomy technique [7,8,9], the one performed during pregnancy has certain different aspects. It involves the division of round ligaments to allow for access to the retroperitoneum, followed by a laborious pelvic lymphadenectomy, which is hampered by the presence of the pregnant uterus. In contrast with the original technique, the preservation of uterine arteries was mandatory to ensure adequate blood supply to the pregnant uterus. Under intraoperative ultrasound guidance, the cervix was carefully sectioned approximately 1 cm away from the amniotic sac, aiming to preserve at least 1 cm of the cervix to ensure contention. Consequently, bilateral parametrectomy with the removal of the parameters, cervix, and upper third of the vagina was performed, followed by cervical cerclage and anastomosis of the vaginal margin to the remaining portion of the cervix.

## 3. Results

Twenty-five cases of abdominal radical trachelectomy performed during pregnancy for early-stage cervical cancer were identified, including five cases at our institute [10,11,12,13,14,15,16,17,18,19,20]. Of those cases, 23 were subjected to the laparotomic approach [10,11,13,14,15,16,17,18,19], and two underwent the laparoscopic approach [12,20]. The most common histological type was squamous cell carcinoma summing up 21 cases (81%) [10,11,14,15,16,17,18,19], with only three cases of adenocarcinoma (12%) [12,19,20] and one case of lymphoepithelial carcinoma (4%) [13]. Twenty procedures (80%) were carried out during the second trimester (14–22 gestational weeks) [11,12,13,14,15,16,17,18,19,20], four (16%) were performed in the first trimester (7–13 gestational weeks) [10], and only one case (4%) was managed during the third trimester (32 weeks of gestation), when a C-section was performed followed by abdominal radical trachelectomy.

Table 1 provides information regarding all abdominal radical trachelectomies performed during pregnancy.

To support the ongoing pregnancy, a cerclage was performed on all patients.

Of the 25 patients included in the study, 19 (81%) gave birth to live newborns through elective C-section, and six patients experienced a miscarriage shortly after the intervention (19%). Among the four patients who had undergone abdominal radical trachelectomy during the first trimester of pregnancy, two miscarried in the first week and one in the third week after the procedure [10]. There were three miscarriages encountered among the 20 patients who underwent the procedure during the second trimester of pregnancy: one case was due to bilateral uterine artery ligation, and extensive pelvic and paraaortic lymphadenectomy leading to fetal death just 4 hours after the intervention [14]; two cases were managed at our clinic due to preterm premature rupture of the membranes. All other pregnant patients (81%) gave birth between 30 and 39 weeks of gestation (average 35.5 weeks) to live newborns [11,12,13,14,15,16,17,18,19,20]. Average operating time was 5.5 h (3.5–8.5 h), and the average amount of blood loss was 1075 mL (200–2510 mL). The minimal volume of blood loss was achieved by the laparoscopic approach [20].

Concerning oncological results, during the follow-up period (6–200 months), no study reported recurrences of the disease.

**Figure 1:**
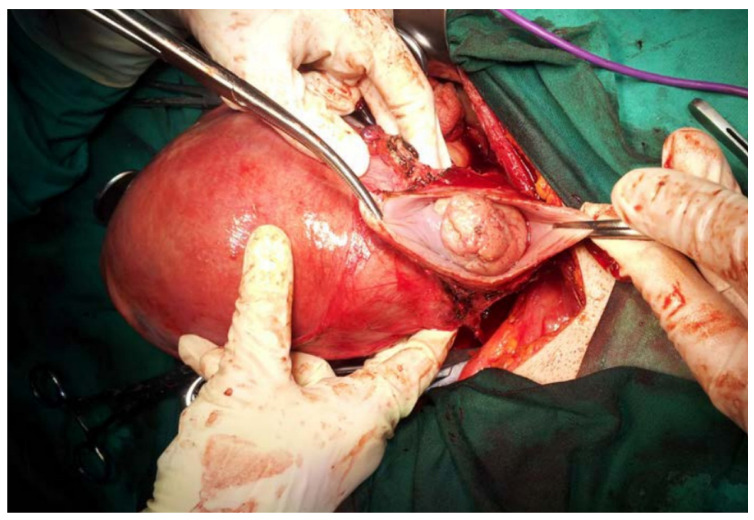
Abdominal radical trachelectomy performed during a 17-week gestation for Stage IB2 cervical cancer.

In the past five years, the authors performed five abdominal radical trachelectomy interventions during pregnancy for early-stage cervical cancer (Stages IB1–IB3, FIGO 2018), out of which three patients (60%) gave birth to live newborns through elective C-section, and two patients (40%) experienced a miscarriage shortly after the intervention (19%). One such case is demonstrated in Figure 1. Four cases were managed in the early second trimester, out of which two miscarried shortly after the procedure due to the preterm premature rupture of membranes. The fifth case of abdominal radical trachelectomy was carried out at the gestational age of 32 weeks and consisted of two steps: first, an elective C-section was performed with the delivery of a live newborn baby, followed by abdominal radical trachelectomy.

Final histological investigation in all five patients showed free surgical margins, with only one case involving a single positive lymph node, necessitating chemotherapy treatment after surgery.

The follow-up period (6–220 months) for patients consisted of regular oncologic evaluation. All patients reached good oncological results.

## 4. Discussion

About 1–3% of women with cervical cancer are diagnosed during pregnancy or postnatally in the first 12 months after delivery [21]. Cervical cancer is also the most common type of cancer encountered in pregnant women with a frequency of 0.8–1.5 cases per 10,000 births [2].

When cervical cancer is diagnosed during pregnancy, it presents an uncertain situation for both clinician and patient, requiring urgent measures. It is commonly agreed that a multidisciplinary approach is needed involving not only gynecological oncologists but also infertility experts, reproductive endocrinologists, and maternal-fetal medicine professionals [22].

The management of cervical cancer during pregnancy must be decided on the basis of the five following aspects: cancer stage, nodal status (if available), histological subtype, gestational age, and the patient’s and her family’s wishes.

Abdominal radical trachelectomy should be offered in centers dedicated to ultraradical surgery, well-experienced in performing radical hysterectomies and other radical interventions in cases of the subsequent identification of advanced cervical cancer during the procedure [23].

Guidelines on the basis of the Third International Consensus Meeting on Gynecologic Cancers in Pregnancy place special importance on the use of neoadjuvant chemotherapy [6]. Most traditional regimes of chemotherapy can be administered from the 14th gestational week and ahead. It is recommended to avoid chemotherapy in the first trimester as it may negatively influence organogenesis.

Data on the pharmacokinetics of chemotherapy during pregnancy are insufficient, and existing statistics are based on a relatively small number of cases. Some large-scale studies assessed the data of children born to mothers who had undergone chemotherapy for extragenital cancers during pregnancy, and showed that the middle- and long-term cognitive and physical outcomes of these children were promising [24,25,26]. However, long-term complications, such as neurodevelopmental impairment, cardiotoxicity, ototoxicity, endocrine disorders, and secondary malignancies, were described [27]. Moreover, there were several reported cases of permanent deafness in children prenatally exposed to platinum-based chemotherapy [28].

Pregnant women receiving neoadjuvant chemotherapy in the second and third trimesters were also at a high risk for prematurity, the preterm premature rupture of membranes, preterm contractions, and low birth weight in up to 50% of infants [29]. Specifically, platinum-based chemotherapy was linked with small-for-gestational-age neonates [24,30].

Early neonatal complications included neonatal death, neonatal intensive care unit admission, small for gestational age, hematologic disturbances, and prematurity-related disorders (respiratory distress syndrome, metabolic disturbances, sepsis, jaundice, and necrotizing enterocolitis). Being small for gestational age may subject the newborns to a high risk of perinatal mortality and morbidity [24,26].

If the patient has a strong desire to preserve the pregnancy and is not willing to expose the fetus to the risks that may arise due to neoadjuvant chemotherapy, then we consider that it is worth granting abdominal radical trachelectomy to appropriately selected cases after properly informing on the risks. The most common complications are miscarriages, chorioamnionitis, the preterm premature rupture of membranes, and preterm deliveries [10,14]. However, in such unfavorable situations, despite the pregnancy loss, the patients’ fertility is preserved.

The abdominal approach has some advantages over the vaginal one. It does not require specialized training because the procedure is similar to a traditional radical hysterectomy, and ideally a more extensive parametrial resection can be achieved [31].

Regarding the effectiveness of the surgical approach in cervical cancer, laparoscopy showed inferior outcomes when compared with open surgery [32,33].

Twenty-four patients included in the current study (96%) underwent an abdominal radical trachelectomy procedure alone, and one patient (4%) underwent a C-section at 32 weeks of gestational age, immediately followed by abdominal radical trachelectomy targeting fertility preservation.

As a rule, it is preferable to avoid abdominal radical trachelectomy in the first trimester of pregnancy [34]. There were documented cases of miscarriages following the procedure during this period, suggesting that fetuses in the first trimester might not be able to proceed to the second trimester. For patients over 20 weeks of gestation, a delay in the treatment until fetal viability was generally accepted. Improved neonatal care allowed for premature delivery with satisfactory outcomes [22].

Sometimes, the patient focuses more on the cancer treatment to the disadvantage of the pregnancy. In such cases, the safety of the mother ought to prevail over the fetus. If the patient chose her own safety, and pregnancy preservation is not aimed for, the treatment of cervical cancer during pregnancy should follow the standard treatment protocol as for nonpregnant patients, maximizing maternal oncological outcome [35].

However, there are some patients who, despite knowing the oncological risks and having appropriate clarification of the narrow knowledge, decided on continuing the pregnancy, opting for the least injurious therapeutic alternative for the fetus. Therefore, they chose to undergo abdominal radical trachelectomy during pregnancy.

The recommendations of the Third International Consensus Meeting on Gynecologic Cancers in Pregnancy [6] approved a less invasive attitude, suggesting a staging lymphadenectomy for Stages IA1 with lymphovascular space invasion, IA2, and IB1 up to the 22nd gestational week as the first step. After the 22nd gestational week, treatment should be postponed until after delivery; alternatively, neoadjuvant chemotherapy should be used to control cancer. In Stage IB2 patients who are at less than 22 weeks of gestation, two options are provided: pelvic lymphadenectomy as an initial step, followed by either chemotherapy or follow up and neoadjuvant chemotherapy following the surgical staging of the illness after downstaging the tumor. If positive nodes are found (including micrometastases), the council recommends the termination of pregnancy. If the subjects refused this possibility, neoadjuvant chemotherapy may be considered after proper counselling about the possible negative outcomes on the prognosis of pregnancy and the lack of existing data. After the 22nd week of gestation, they argue that only neoadjuvant chemotherapy is an option. The same rules applied to Stage IB3 [6].

However, the above-mentioned guidelines [6] did not advise the use of abdominal radical trachelectomy during pregnancy for early-stage cervical cancer on the basis of a few cases reporting significant blood loss and prolonged procedure duration. Currently, together with the authors’ cases, there were 25 abdominal radical trachelectomies that were performed during pregnancy. Among them, 81% (19 patients) had good obstetric results, giving birth through elective C-section to well-adapted newborns, and only 19% (6 patients) had an unfortunate outcome, miscarrying shortly after the abdominal radical trachelectomy procedure. Ungar et al. reported three miscarriages among abdominal radical trachelectomies performed in the first trimester of pregnancy (7–13 gestational weeks). Two of these miscarriages were on the first and one was on the 17th day following the procedure [10]. Karateke et al. reported a 22 gestational week intrauterine fetal death four hours after the procedure [14]. The regrettable event might have occurred owing to the ligation of both uterine arteries. The autopsy revealed no fetal anomalies, only hypoxic alterations of the placenta were noted, suggesting insufficient blood flow by the ovarian arteries alone during advanced pregnancy [14]. Additionally, two of our five cases resulted in a miscarriage. Both procedures were carried out in the second trimester of pregnancy (15 gestational weeks) for Stage IB3 cervical cancer, and preterm premature rupture of membranes occurred on the seventh and eighth day, respectively, following the surgical procedure, subsequently leading to a miscarriage.

When the preservation of a pregnancy is the aim, gynecologic oncologists face a difficult situation. They must balance the mother’s safety by achieving the best results in terms of oncological outcomes (tumor-free surgical margins, if possible) with the continuation of the pregnancy and the wish of preserving future fertility. Fertility preservation is essential, as there is a probability for the ongoing pregnancy to end in a miscarriage following the abdominal radical trachelectomy.

Long-term data regarding oncological and obstetric outcomes following abdominal radical trachelectomy performed during pregnancy are limited [36]. Assuming the surgical procedure of abdominal radical trachelectomy during pregnancy is about the same as the abdominal radical trachelectomy in nonpregnant patients, it is presumed that the long-term outcomes are about the same. Gizzo et al. analyzed 1293 patients who underwent a radical trachelectomy, pointed out a disease recurrence risk of 3%, and stated that most patients became pregnant spontaneously following the radical trachelectomy [37]. Consequently, abdominal radical trachelectomy appears to be a safe option for eligible women with good oncological outcomes who wish to retain their fertility and preserve the ongoing pregnancy [38].

The most common pregnancy complications are miscarriages, chorioamnionitis, preterm premature rupture of the membranes, and preterm deliveries [10,14]. One of the explanations for obstetric complications might be the shortening of the cervix following the procedure, compromising its mechanical support and protective function against ascending infections. Kasuga et al., in their study regarding pregnancies following abdominal radical trachelectomy, discovered that patients with a short residual cervical length of <13 mm in the mid-trimester (measured with transvaginal ultrasonography) had a higher risk of delivery before 34 gestational weeks [39].

Despite the preoperative measurement of the length between cervix and fetal membranes, while performing the abdominal radical trachelectomy for early bulky cervical cancers, the desire to acquire the safest oncological result (tumor-free surgical margins) may compromise the integrity of the amniotic membranes, subsequently leading to the premature rupture of the membranes. The authors recommended taking special care considering this aspect.

Intraoperative approximation of trachelectomy margins was applied using the transverse and perpendicular method [40]. To support the ongoing pregnancy, a cerclage was performed on all patients.

Particular caution was applied regarding vascular anatomical variations that were a source of acute perioperative bleeding [41].

Concerning oncological results, during the follow-up period (6–200 months) consisting of regular oncological evaluations, no study reported recurrences of the disease. The authors did not signal the presence of recurrences of the disease during the follow-up period of the patients they operated on.

This study urges awareness of the fact that abdominal radical trachelectomy performed during pregnancy is an intervention that can be proposed to patients opposed to the idea of neoadjuvant chemotherapy. If the patients have a strong desire to preserve the pregnancy and are not willing to expose the fetus to the risks that may arise due to neoadjuvant chemotherapy, it is worth attempting abdominal radical trachelectomy to appropriately selected cases after considering proper information regarding the risks.

This study contained the highest number of abdominal radical trachelectomy cases performed during pregnancy for early-stage cervical cancer, including five cases performed by the authors, which were not previously published. The procedures were carried out in different countries and centers, thus offering a broader perspective on the effectiveness of this niche intervention. The limitations reside in its retrospective manner and the lack of available data regarding this subject. However, to clarify the efficiency of this procedure, future studies are mandatory.

Sharing our clinical experience with an international registry, such as The International Network on Cancers, Infertility, and Pregnancy (www.cancerinpregnancy.org), and the European Society of Gynecological Oncology, is a worthwhile endeavor [18].

## 5. Conclusions

Abdominal radical trachelectomy may be considered for appropriately selected patients with early-stage cervical cancer who have a strong desire to preserve their pregnancy and who are not willing to expose the fetus to the risks associated with neoadjuvant chemotherapy. The early second trimester of pregnancy appears to be the most suitable period to carry out the procedure.

Cervical cancer in young pregnant patients is a dire situation that can endanger both patient and fetus. Patient fertility is also endangered. These patients must be treated with exceptional care in specialized centers, where a multidisciplinary team can offer them the most efficient therapeutic strategies. Currently, neoadjuvant chemotherapy is considered to be the safest therapeutic option. Thus, abdominal radical trachelectomy does not represent the current standard of care for cervical cancer during pregnancy. However, the results of the current study were satisfactory, and in carefully selected cases, the therapeutic alternative of an abdominal radical trachelectomy provided an immediate solution, offering a chance to both the mother and the unborn baby. More evidence on its effectiveness must be provided.

## Figures and Tables

**Table 1:** Abdominal radical trachelectomy performed during pregnancy—a review of the literature and our institutional experience.

Publication	Gestational Age at Surgery, wk.	Stage	Histology	Status of Uterine Arteries	Duration of Surgery	Estimated Blood Loss, mL	Pregnancy Outcome, wk.	Neonatal Outcome	Oncological Outcome
**Abdominal Radical Trachelectomy**									
Ungar et al., 2006 [10]	7	IB1	SCC	Both preserved	NR	NR	Mis.—1st wk. after surgery	-	NED
Ungar et al., 2006 [10]	8	IB1	SCC	Both preserved	NR	NR	Mis.—1st wk. after surgery	-	NED
Ungar et al., 2006 [10]	9	IB1	SCC	Both preserved	NR	NR	38 wk.	Good	NED
Ungar et al., 2006 [10]	13	IB1	SCC	Both preserved	NR	NR	Mis.—3rd wk. after surgery	-	NED
Ungar et al., 2006 [10]	18	IB1	SCC	Both preserved	NR	NR	39 wk.	Good	NED
Mandic et al., 2009 [11]	19	IB1	SCC	NR	5 h	450	36 wk. (PROM)	Good	NED
Abu-Rustum et al., 2009 [13]	15	IB1	Lymphoepithelial	Left ligature	3.5 h	1600	39 wk.	Good	NR
Karateke et al., 2010 [14]	22	IB2	SCC	Bilateral ligature	4 h	200	Mis.—fetal death a few hours after surgery	-	NR
Enomoto et al., 2011 [15]	15	IB1	SCC	Right ligature	7.5 h	960	37 wk.	Good	NED
Aoki et al., 2014 [16]	17	IB1	SCC	Both preserved	6 h	2510	38 wk.	Good	NED
Căpîlna et al., 2015 [17]	17	IB2	SCC	Left ligature	6 h	500	38 wk.	Good	NED
Căpîlna et al., 2016	15	IB3	SCC	Both preserved	5.5 h	800	Mis.—2nd wk. after surgery (PPROM)	-	NED
Căpîlna et al., 2016	15	IB3	SCC	Both preserved	6 h	500	Mis.—2nd wk. after surgery (PPROM)	-	NED (CRT following surgery)
Căpîlna et al., 2016	17	IB2	SCC	Left ligature	5 h	500	39 wk.	Good	NED
Căpîlna et al., 2019	32 (C-section + ART)	IB3	SCC	Both ligation	5 h	900	32 wk.	Good	NED
Rodolakis et al., 2018 [18]	14	IB1	SCC	Both preserved	4.5 h	1800	36 wk.	Good	NED
Rodolakis et al., 2018 [18]	14	IB1	SCC	Both preserved	4 h	2000	32 wk.	Good	NED
Yoshihara et al., 2018 [19]	15	IB1	SCC	Both preserved	8.5 h	1275	33 wk. (PROM)	Good	NED (CT following surgery)
Yoshihara et al., 2018 [19]	17	IB1	SCC	Left ligature	6.5 h	600	37 wk.	Good	NED
Yoshihara et al., 2018 [19]	15	IB1	SCC	Both preserved	6 h	1270	30 wk.	Good	NED (CT following surgery)
Yoshihara et al., 2018 [19]	15	IB1	SCC	Bilateral ligature	5 h	935	33 wk.	Good	NED (CT following surgery)
Yoshihara et al., 2018 [19]	15	IB1	AC	Both preserved	5.5 h	1415	37 wk.	Good	NED
Yoshihara et al., 2018 [19]	17	IB1	SCC	Left ligature	6.5 h	2010	31 wk.	Good	NED (CT following surgery)
**Laparoscopic Abdominal Radical Trachelectomy**									
Kyrgiou et al., 2015 [20]	14	IB1	AC	Both preserved	4 h	200	36 wk.	Good	NED
Yi et al., 2015 [12]	18	IB1	Papillary mucinous AC	N/A	N/A	N/A	34 wk.	Good	N/A

Wk, week; SCC, squamous cell carcinoma; AC, adenocarcinoma; Mis, miscarriage; NR, not reported; N/A, not available; NED, no evidence of disease; PROM, premature rupture of membranes; PPROM, preterm premature rupture of membranes; CRT, chemoradiotherapy; CT, chemotherapy.

## Data Availability

We provide our data for the reproducibility of this study in other centers if such is requested.
